# Retraction statement: Lymphocyte‐specific protein 1 inhibits the growth of hepatocellular carcinoma by suppressing ERK1/2 phosphorylation

**DOI:** 10.1002/2211-5463.13509

**Published:** 2022-11-15

https://doi.org/10.1002/2211-5463.12139

The above article published online on 06 October 2016 in Wiley Online Library (wileyonlinelibrary.com), has been retracted by agreement between the Editor‐in‐Chief and John Wiley and Sons Ltd. The retraction has been agreed following an investigation based on allegations raised by a third party, which revealed inappropriate and unexplained duplications between this and another article [1]. Thus, the editors consider the conclusions of this manuscript substantially compromised.
